# Origin and Dissemination of Altered El Tor Vibrio cholerae O1 Causing Cholera in Odisha, India: Two and Half Decade’s View

**DOI:** 10.3389/fmicb.2021.757986

**Published:** 2021-11-18

**Authors:** Bibhuti Bhusan Pal, Dipti Ranjan Behera, Smruti Ranjan Nayak, Ashish Kumar Nayak

**Affiliations:** Microbiology Division, ICMR-Regional Medical Research Centre, Bhubaneswar, India

**Keywords:** origin, spread, V. cholerae O1, ctxB1 and ctxB7 genotypes, Odisha

## Abstract

The origin, spread and molecular epidemiology of altered El Tor Vibrio cholerae O1 strains isolated from cholera outbreaks/surveillance studies between 1995 and 2019 from different district of Odisha were analyzed. The stock cultures of V. cholerae O1 strains from 1995 to 2019 were analyzed through molecular analysis using different PCR assays and pulse field gel electrophoresis (PFGE) analysis. The spread map (month, year and place) was constructed to locate the dissemination of altered El Tor variants of V. cholerae O1 in this region. A total of 13 cholera outbreaks were caused by V. cholerae O1 Ogawa biotype El Tor carrying ctxB1 and ctxB7 genotypes. The ctxB1 alleles of V. cholerae O1 mostly confined to the coastal areas, whereas the ctxB7 genotypes, though originating in the coastal region of Odisha, concentrated more in the tribal areas. The positive correlation between virulence-associated genes (VAGs) was found through Pearson’s correlation model, indicative of a stronger association between the VAGs. The clonal relationship through PFGE between ctxB1 and ctxB7 genotypes of V. cholerae O1 strains exhibited 80% similarity indicating single- or multi-clonal evolution. It is evident from this study that the spread of multidrug-resistant V. cholerae O1-altered El Tor was dominant over the prototype El Tor strains in this region. The origin of altered El Tor variants of V. cholerae O1 occurred in the East Coast of Odisha established that the origin of cholera happened in the Gangetic belts of Bay of Bengal where all new variants of V. cholerae O1 might have originated from the Asian countries.

## Introduction

Cholera is a severe form of watery diarrheal disease, dates back to antiquity, and is caused by the ingestion of food or water contaminated with the pathogenic strains of Vibrio cholerae of serogroup O1 or O139 (Pollitzer, 1954). Vibrio cholerae is an autochthonous inhabitant of the estuarine aquatic environment of the Bay of Bengal (Alam et al., 2006), where low salinity of rivers or shallow wells (avg. 2.8–8.2 ppt) and temperatures between 26°C and 35°C during the dry season favors the growth and multiplication of V. cholerae (Moore et al., 2014). V. cholerae has evolved into more pathogenic types due to various genetic assortments and re-assortments in the core toxin region which gives rise to altered and hybrid variants of prototype V. cholerae strains (Faruque et al., 2004), causing higher mortality with recorded 21,000–143,000 deaths worldwide (Ramamurthy et al., 2019). The biotype El Tor of V. cholerae O1 has been changing the whole disease scenario perpetually due to its better survival capacity in the environment as well as in the human host and is able to produce significantly higher amounts of cholera toxin (CT) in vivo (Ghosh-Banerjee et al., 2010; Grim et al., 2010). Among different El Tor variants, the altered El Tor V. cholerae O1 strains have prevailed in different regions of the world, including the US Gulf coast and several countries of Asia and Africa (Nair et al., 2006; Goel et al., 2010; Pal et al., 2017). Odisha, situated at the eastern coast of India, has recorded several cholera outbreaks/epidemics. The cholera epidemics after the disastrous super cyclone in 1999 affected more than 10 million of the population. This might be due to coastal saline-rich aquatic environmental water along the side of Bay of Bengal favoring V. cholerae to spread its territory. Cholera has been reported from Odisha over the last two and half decades (Chhotray et al., 2002; Khuntia et al., 2010; Pal et al., 2010, 2017). However, no detailed molecular epidemiological report is available on the origin and dissemination of the altered El Tor V. cholerae O1 strains to different parts of Odisha. In this study, we have undertaken a retrospective analysis on V. cholerae O1 strains which were isolated during the cholera outbreaks and surveillance studies reported from 1995 to 2019 from different districts of Odisha to illustrate the chronology of the appearance and spread of V. cholerae O1 El Tor variant strains to different parts of the state.

## Materials and Methods

### Revival of Strains

A total of 1,492 strains of V. cholerae O1 isolated from the coastal and tribal areas of Odisha from 1995 to 2019 during cholera outbreaks and surveillance studies were included in this study (Table 1). Thiosulfate–citrate–bile–salt–sucrose (TCBS) agar was used to isolate the V. cholerae strains isolated from stool and environmental water samples. Big moist yellow colonies from TCBS agar plates were selected for different biochemical tests (Pal et al., 2010), and serotyping was done by slide agglutination tests with polyvalent O1 and monospecific Ogawa, Inaba antisera (BD, San Jose, CA, United States). In addition, isolated colonies of V. cholerae strains from TCBS agar plates were re-streaked on Luria Bertani agar (BD, United States), incubated at 37°C for 24 h and then inoculated in Luria Bertani broth (BD, United States), kept in a shaker incubator at 37°C overnight for bacterial growth, and subsequently used for DNA isolation using the boiling method (Pal et al., 2017).

**TABLE 1:** Year-wise isolation of Vibrio cholerae O1 strains from different districts of Odisha: 1995–2019.

Year	No of strains	V. cholerae serogroup	Isolation places (district)
1995	16	O1 Ogawa	Cuttack
1996	7	O1 Ogawa	Cuttack
1997	17	O1 Ogawa	Cuttack
1999	65	O1 Ogawa	Cuttack, Jagatsinghpur, Puri, Kendrapara, Jajpur, Balasore, Bhadrak
2000	87	O1 Ogawa	Jagatsinghpur, Puri, Berhampur, Jajpur
2001	58	O1 Ogawa	Puri, Kendrapara, Jajpur, Bhadrak, Balasore, Khordha
2002	82	O1 Ogawa	Puri, Gajapati, Rayagada
2003	160	O1 Ogawa	Puri, Dhenkanal, Keonjhar, Mayurbhanj, Malkangiri
2004	47	O1 Ogawa/Inaba	Puri, Khordha, Dhenkanal
2005	86	O1 Ogawa/Inaba	Cuttack, Puri, Khordha, Kendrapara, Dhenkanal
2006	62	O1 Ogawa/Inaba	Cuttack, Puri, Kendrapara
2007	166	O1 Ogawa/Inaba	Jagatsinghpur, Khordha, Rayagada, Koraput
2008	128	O1 Ogawa/Inaba	Cuttack, Jagatsinghpur, Khordha, Puri, Sundergarh
2009	71	O1 Ogawa	Puri, Khordha, Kendrapara, Mayurbhanj, Rayagada, Kalahandi, Sundergarh
2010	67	O1 Ogawa	Gajapati, Rayagada, Kalahandi
2011	57	O1 Ogawa	Rayagada, Gajapati
2012	153	O1 Ogawa	Rayagada, Kalahandi, Koraput
2013	96	O1 Ogawa	Rayagada, Koraput
2014	11	O1 Ogawa	Kalahandi
2015	1	O1 Ogawa	Nuapada
2016	4	O1 Ogawa	Balasore, Rayagada, Nuapada
2018	5	O1 Ogawa	Bargarh, Rayagada, Kalahandi
2019	31	O1 Ogawa	Rayagada
Total	1492		

### Multiplex PCR Assays

All phenotypically confirmed V. cholerae isolates were further confirmed by multiplex PCR (mPCR) assays by targeting the genes of V. cholerae, such as species-specific gene ompW, outer membrane protein (ompU) gene, identifying genes encoding O1 (rfbO1) and O139 (rfb O139), and major virulence and toxic genes ctxA, tcpA, ace, toxR, and rtxC (Kimsey and Waldor, 1998; Nandi et al., 2000; Chow et al., 2001).

### Analysis of Virulence Genes

The presence/absence of eight virulence-associated genes in each isolate obtained from 1995 to 2019 was used to define the virulence-associated gene profile. The profile was developed by PCR analysis by calculating the percentage of virulence-associated genes present in each strain of V. cholerae O1. The profiles of all isolates were analyzed by hierarchical clustering using a complete linkage method. The dendrogram and a heat map were constructed using the PAST 4.03 software and GraphPad Prism 7 statistical package, respectively. Pearson’s correlation coefficient analysis was employed to identify the correlation between the virulence genes (Prajapati et al., 2020).

### ctxB Genotyping

Mismatch amplification mutation assay (MAMA) and double-mismatch amplification mutation assay (DMAMA) PCR reactions were used to detect the type of ctxB in all the laboratory stocks of V. cholerae O1 strains isolated during cholera outbreaks and surveillance studies from 1995 to 2019 (Morita et al., 2008; Naha et al., 2012).

### Pulsotyping by Pulsed-Field Gel Electrophoresis

Pulsotyping by pulsed-field gel electrophoresis (PFGE) was performed on some selected strains of V. cholerae O1 isolated from 1995 to 2019. The DNA sample of V. cholerae O1 in agarose plugs was digested with 50 U of NotI (New England Biolabs, Ipswich, MA, United States). The digested DNA was separated through 1% agarose gel (Bio-Rad, Hercules, CA, United States) in 0.5× TBE buffer (pH 8.4) at 14°C in a CHEF Mapper system (Bio-Rad, United States). The dendrograms were constructed on the basis of banding similarity and dissimilarity using the Dice coefficient, and clustering was based on the unweighted pair group method with arithmetic mean (UPGMA) with a band position tolerance of 1.2% (Cooper et al., 2006).

## Results

### Distribution of V. cholerae O1 Strains in Endemic Districts

**FIGURE 1:**
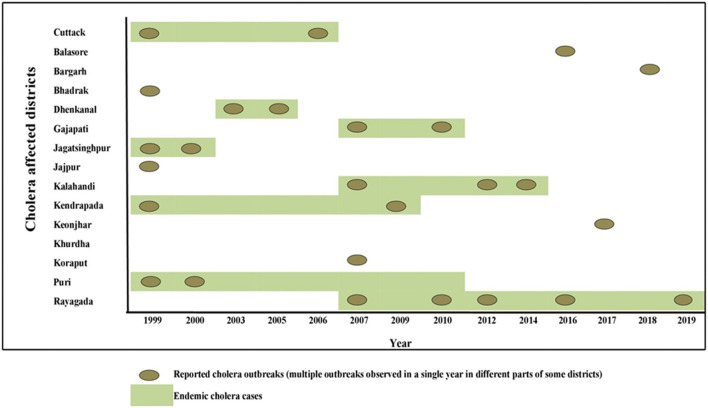
Distribution of cholera cases and outbreaks in Odisha: 1999–2019.

Odisha, situated at the eastern coast of India, has encountered 13 cholera outbreaks from 1999 to 2019. Details of the epidemiological data of these cholera outbreaks are depicted in Table 2. It is evident that mostly coastal districts, namely, Cuttack, Jagatsinghpur, Puri, Kendrapara, Jajpur, Balasore, and Bhadrak, were the most affected districts due to cholera in Odisha. Later, the disease was transmitted to the tribal districts of Odisha, namely, Rayagada, Koraput, Kalahandi, Gajapati, and Keonjhar (Figure 1). There were more than 3 million people at risk for cholera in these five tribal districts of Odisha, where cholera has become endemic with repeated waves of outbreaks that occurred in the recent past. The average incidence rate of cholera cases in these endemic areas is 39.89 cases/1,000 population at risk per year.

**TABLE 2:** Antibiotic resistance profiles with ctxB genotypes of Vibrio cholerae O1 associated with different cholera outbreaks in Odisha: 1999–2019.

Outbreakyear	Affecteddistricts	Population affected	Cases reported	Incidence rate (IR)/1,000 individual	Serotypes(%)	Antibiogramresistanceprofile	Genotypes
1999	Cuttack, Jagatsinghpur, Kendrapara, Jajpur, Puri, Bhadrak, Balasore	8,043,000	97,934	12.18	O1 (Ogawa) = 72.3 O139 = 7.2	AFrCoSNaN	ctxB1
2000	Jagatsinghpur, Puri	2,200,000	198	0.09	O1 (Ogawa) = 58.6 O139 = 40.2 Non O1/O139 = 1.2	FrCoNaN	ctxB1
2003	Dhenkanal	946	41	43.34	O1 (Ogawa) = 66.7	AFrCoSNa	ctxB1
2005	Dhenkanal	2,102	113	53.76	O1 (Ogawa) = 22.2 O1 (Inaba) = 66.7	FrNa	ctxB1
2006	Cuttack	10,621	146	13.75	O1 (Ogawa) = 82.6	FrCoSNa	ctxB1
2007	Rayagada, Koraput, Kalahandi, Gajapati	123,546	8,206	66.42	O1 (Ogawa) = 94.9 O1 (Inaba) = 2.6	AmFrSNNfNaCo	ctxB1
2009	Kendrapara (Rajnagar), Mayurbhanj	108,500	809	7.45	O1 (Ogawa) = 67	AmFrCoCCfSNa	ctxB7
2010	Rayagada, Gajapati	113,375	2,152	18.98	O1 (Ogawa) = 51.5	AmTeNaFrSECoNC	ctxB1
2012	Rayagada, Kalahandi	735,647	641	0.87	O1 (Ogawa) = 42	AmNaFrSCo	ctxB1, ctxB7
2014	Kalahandi	46,236	321	6.94	O1 (Ogawa) = 64.7	AmFrNaNECGCo	ctxB7
2016	Balasore, Rayagada	59,937	138	2.30	O1 (Ogawa) = 88.9	CFrCoSNaN	ctxB7
2018	Bargarh	1,387	55	39.65	O1 (Ogawa) = 13.3	AFrCoSNa	ctxB7
2019	Rayagada	500	73	146.00	O1 (Ogawa) = 50 Non O1/O139 = 7.1	AFrCoSNaN	ctxB7
	Total	1,145,0199	110,894				

### Genotypic Characteristics of Cholera Strains

**FIGURE 2:**
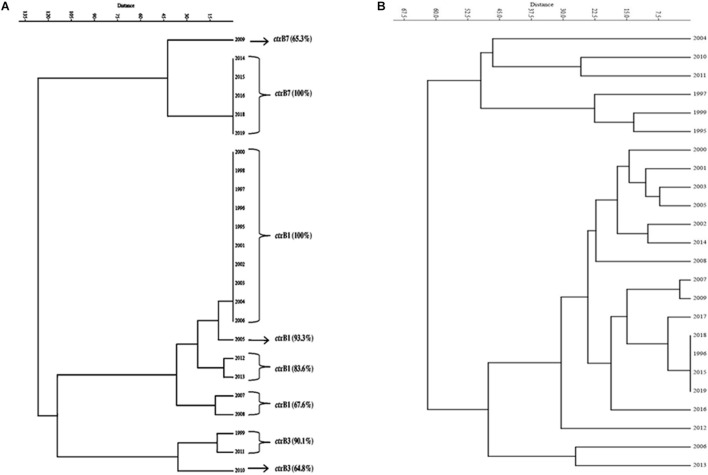
Dendrogram drawn using PAST 4.03 software. **(A)** Cluster analysis of the estimate of genetic similarity between ctxB genotypes present in V. cholerae O1 strains in different years. **(B)** Cluster analysis of the estimate of percentage similarity of virulence-associated genes (VAGs) present in V. cholerae O1 strains in different years.

A total of 1,492 V. cholerae strains isolated from 1995 to 2019 were serologically confirmed to be V. cholerae O1. During this study period, V. cholerae O1 strains belonging to serotype Ogawa exhibited dominance (92.4%) over serotype Inaba (7.6%) and each strain of V. cholerae O1 was positive for the cholera toxin gene, i.e., ctxA. In addition, El Tor biotype-specific toxin co-regulated pilus (tcpA^ET^) showed dominance from 1995 to 2006, in contrast to tcpA^Haitian^ type strains that showed dominance from 2007 to 2019. All the V. cholerae O1 strains also showed positive results for virulent and accessory genes such as ompW, ompU, rtxC, toxR, and ace that regulate the toxin production of V. cholerae. From the MAMA PCR assay, it was evident that the El Tor ctxB3 genotype of V. cholerae O1 emerged in 1999 during the super cyclone, became quiescent up to 2004, then increased gradually from 2005 to 2011 (except in 2006) and subsequently disappeared. The DMAMA PCR assay revealed that V. cholerae O1 strains isolated from 1995 to 2012 including outbreak strains possessed the ctxB1 genotype (classical type CT) except the cholera outbreak in 2009 that happened due to the ctxB7 genotype (Haitian type CT). Between 2012–2019, the outbreak strains with the Haitian ctxB7 allele circulated and predominated within the different tribal districts of Odisha (Table 2). The hierarchical clustering analyses of ctxB alleles isolated in different time periods from 1995 to 2019 showed three separate clusters indicative of three different lineages with significant relatedness distributed among the strains of V. cholerae O1 (Figure 2A).

### Profiling of Virulence-Associated Genes

**FIGURE 3:**
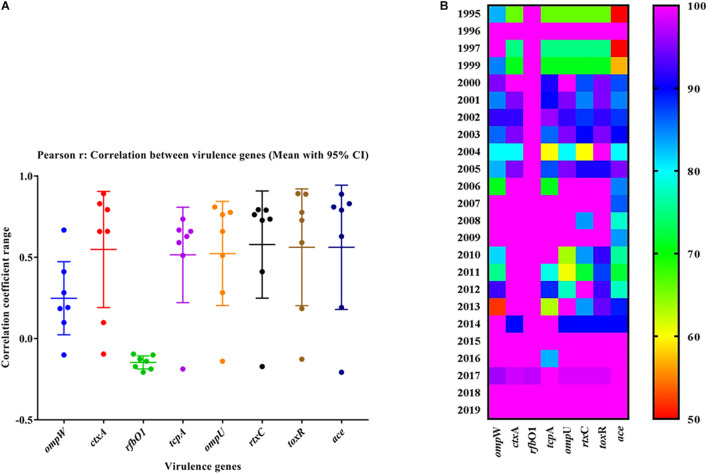
**(A)** Scatter plot of the correlation between virulence genes of Vibrio cholerae O1 strains. The data represent different magnitudes of correlation coefficient (r) ranges between –0.5 and 1.0. A positive correlation indicated a stronger association between the variables and vice versa. The greater the absolute value of the Pearson product–moment correlation coefficient, the stronger the linear relationship. **(B)** Heat map showing the percentage variation in different virulence-associated genes distributed among the strains in different years.

The mPCR assay on virulence-associated genes (VAGs) on 306 randomly selected V. cholerae O1 strains showed variation ranging from 50 to 100% as indicated by different color intensities in a heat map. The correlation analysis showed a significant positive correlation between VAGs except for the somatic O-antigen biosynthesis gene (rfbO1) which showed a negative correlation with other VAG genes such as outer-membrane protein gene (ompW), cholera toxin gene (ctxA), toxin co-regulated pilus gene (tcpA), outer-membrane protein gene (ompU), repeat in toxin protein (rtxC), toxin regulator gene (toxR), and the accessory cholera enterotoxin gene (ace). Pearson’s correlation coefficient values of each VAGs are presented in Table 3. The scatter plot for correlation values and the heat map are presented in Figures 3A,B, respectively. Hierarchical clustering based on VAGs identified in different strains from 1995 to 2019 showed discrete clusters indicative of insignificant relatedness between percentages of VAGs present in different years or their non-uniform distribution among the V. cholerae O1 strains (Figure 2B).

**TABLE 3:** Correlation between virulence genes of Vibrio cholerae O1 strains using Pearson’s correlation coefficient model.

	ompW	ctxA	rfbO1	tcpA	ompU	rtxC	toxR	ace
ompW	1							
ctxA	0.09	1						
rfbO1	−0.11	−0.08	1					
tcpA	0.71	0.58	−0.19	1				
ompU	0.28	0.57	−0.13	0.41	1			
rtxC	0.42	0.74	−0.17	0.68	0.71	1		
toxR	0.19	0.84	−0.12	0.49	0.72	0.65	1	
ace	0.22	0.76	−0.22	0.54	0.77	0.74	0.84	1

### Spread of Altered El Tor V. cholerae O1

#### Wave of Cholera Toxin Genotype 1 (Classical Cholera Toxin)

**FIGURE 4:**
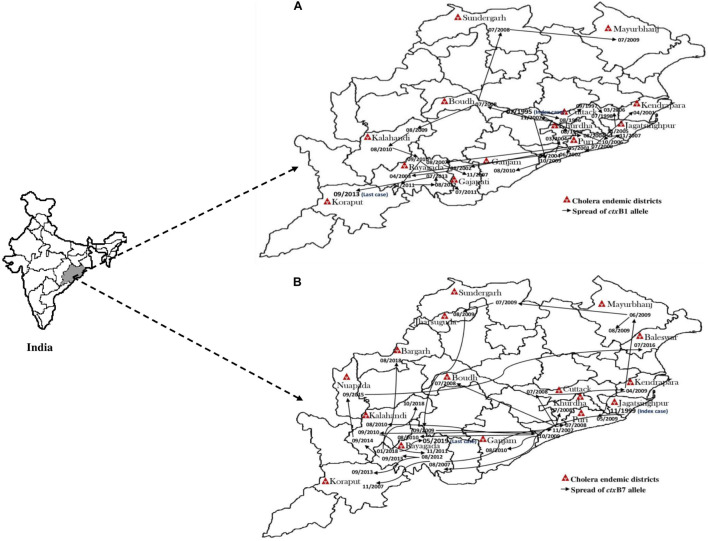
Month- and year-wise spread of El Tor variant *Vibrio cholerae* O1 strains. **(A)** Spread of *ctx*B1 **(B)** spread of *ctx*B7 genotypes in Odisha: 1995–2019.

The El Tor variant strains of V. cholerae O1 carrying classical CT genotype 1 (ctxB1) emerged in the coastal regions of Odisha in 1995. The first case was reported from Cuttack in July 1995. Out of 25 V. cholerae isolates, 23 were V. cholerae O1 Ogawa biotype El Tor carrying classical CT, and 3 were V. cholerae O139 serogroup. The gradual dissemination of the ctxB1 allele of V. cholerae O1 in the coastal districts, namely, Jagatsinghpur, Kendrapara, Puri, Balasore, Bhadrak, and Jajpur, occurred in successive years and propounded into large cholera outbreaks in 1999, 2000, 2003, 2005, 2006, 2007, 2010, and 2012 with 109,431 reported cholera cases (Table 2). The El Tor variant strains carrying classical ctxB spread to the tribal districts of Odisha in 2007. The first case was reported in August 2007 in the Kashipur block of Rayagada district, subsequently spread to adjacent districts in successive years. The detailed outline of the spread of the ctxB1 allele of El Tor variant V. cholerae O1 is predicted in Figure 4A.

#### Wave of Cholera Toxin Genotype 7 (Haitian Cholera Toxin)

**FIGURE 5:**
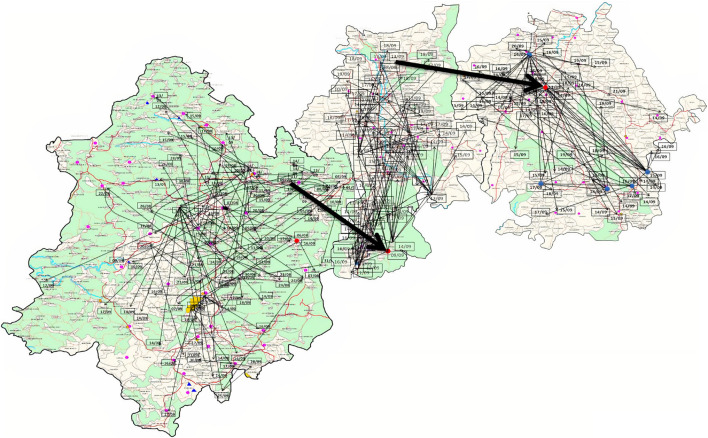
Spread of *V. cholerae* O1 biotype El Tor in three blocks of Rayagada district (Kashipur, Kalyansinghpur, and Bissam Cuttack): August–September, 2010.

A new variant of the El Tor biotype of V. cholerae O1-producing Haitian cholera toxin (Haitian CT) emerged from the super cyclone of Odisha in October 1999. The first cholera case possessing ctxB genotype 7 was reported from the Erasama block of Jagatsinghpur district in November 1999. Later, the Haitian variant strains reemerged from the tribal areas of Rayagada and Koraput districts in August and November 2007 and then spread to coastal areas of Puri in November 2007. The outbreaks continued in the successive years by ctxB7 genotypes of V. cholerae O1 to neighboring coastal districts, namely, Cuttack, Khordha, and Puri, in July 2008. Later, the ctxB7 genotypes of V. cholerae O1 spread and caused large cholera outbreaks in Kendrapara and Mayurbhanj districts in 2009 by affecting 104,327 populations with 783 reported cases. Subsequently, the transmission of Haitian variant V. cholerae O1 strains was confined to the tribal areas of Odisha particularly in Rayagada, Kalahandi, and Koraput districts. The spread map of the ctxB7 allele of V. cholerae O1 biotype El Tor is shown in Figure 4B. This showed that the HCT variant strains of V. cholerae O1 have spread predominantly to the eastern and southern parts of Odisha in 2010, 2011, 2012, 2014, 2016, and 2018 and to a lesser extent to western and northern parts of Odisha in 2019. The month- and village-wise spread of cholera cases during the 2010 cholera outbreak in three tribal blocks, i.e., Kashipur, Kalyansinghpur, and Bissam Cuttack of Rayagada district, due to HCT variant V. cholerae O1 Ogawa has been described, which is very interesting (Figure 5).

### Severe Cholera Cases Among Tribal Populations

The spread of altered El Tor strains of *V. cholerae* O1 among the tribal areas has the connecting link with the coastal outbreak strains, evident through the sequential occurrence of cholera cases. In total, 520 *V. cholerae* O1 strains were collected from the tribal areas of Odisha from 2002 to 2019 from Rayagada, Kalahandi, Koraput, Gajapati, Keonjhar, Sundergarh, Mayurbhanj, Nuapada, and Boudh (Table 4). The first appearance of hybrid El Tor strains carrying classical CT was reported from the Mohana block of Gajapati district in 2002. This hybrid strain of *V. cholerae* O1 emerged as an epidemic form in August 2007 in the four tribal districts, namely, Rayagada, Gajapati, Kalahandi, and Koraput, by affecting 358 villages with a population at risk of 123,546 (Pal et al., 2010). The altered El Tor strains of *V. cholerae* remained in the environmental reservoirs and reemerged as epidemic strains in 2010 in the tribal areas of Rayagada and Gajapati districts causing cholera outbreaks, accounting for 2,152 cholera cases. Interestingly, all the isolates of *V. cholerae* O1 showed resistance to tetracycline, a unique feature first reported from cholera outbreaks in the tribal areas of Odisha (Kar et al., 2015). The cholera infection again returned to some parts of Rayagada and Kalahandi in 2012 due to a mixed infection of *ctx*B1 and *ctx*B7 alleles of *V. cholerae* O1 Ogawa biotype El Tor, which accounted for 641 cholera cases. The first case of the Haitian variant (*ctx*B7 genotype) strain of *V. cholerae* O1 from tribal areas was obtained from the cholera outbreak in 2009 from Mayurbhanj district with a total of 94 cases which were reported with one death (CFR—1.06%). Subsequently, the spread of the *ctx*B7 allele of *V. cholerae* O1 in the tribal areas was reported from the Kalahandi district in 2014, then from Rayagada and Balasore districts in 2016, and reappeared in a recent cholera outbreak from Rayagada district in 2019. A total of 575 cholera cases were reported from these cholera outbreaks with an incidence rate of 40.29% and a case fatality rate (CFR) of 10.13%. The common resistance profile of isolated *V. cholerae* O1 strains obtained in these outbreaks was ampicillin, nalidixic acid, furazolidone, streptomycin, neomycin, erythromycin, and co-trimoxazole (Pal et al., 2017, 2021; Nayak et al., 2020).

**TABLE 4:** Year-wise isolation of *Vibrio cholerae* O1 strains from the tribal areas of Odisha: 2002–2019.

Year	Block/Town	District	Number of strains isolated	Strain type (genotypes)
2002	1. Kashipur 2. Mohana	1. Rayagada 2. Gajapati	21	El Tor variant strains of *V. cholerae* (*ctx*B1)
2007	1. Kashipur 2. Dasamantapur	1. Rayagada 2. Koraput	51	El Tor variant strains of *V. cholerae* (*ctx*B1 and *ctx*B7)
2008	1. Boudh	1. Boudh	12	El Tor variant strains of *V. cholerae* (*ctx*B1 and *ctx*B7)
2009	1. Karanjia, Pandra, Sindurgoura, Badakuldhia 2. Biswanathpur 3. Koira 4. Kashipur	1. Mayurbhanj 2. Kalahandi 3. Sundergarh 4. Rayagada	25	El Tor variant strains of *V. cholerae* (*ctx*B1 and *ctx*B7)
2010	1. Kashipur, Kalyansingpur, Bissam Cuttack 2. Mohana	1. Rayagada 2. Gajapati	66	Typical El Tor strains of *V. cholerae* (*ctx*B3) El Tor variant strains of *V. cholerae* (*ctx*B1)
2011	1. Kolnara, Bissam Cuttack 2. Mohana, R.Udayagiri, Govindpur, Liligad, Adora, Sikulipadar	1. Rayagada 2. Gajapati	54	Typical El Tor strains of *V. cholerae* (*ctx*B3) El Tor variant strains of *V. cholerae* (*ctx*B1)
2012	1. Kashipur, Kalyansingpur, Gudari, Gunupur, Rayagada 2. Kalampur, Jaipatna, Junagarh 3. Laxmipur, Dasamantapur	1. Rayagada 2. Kalahandi 3. Koraput	153	El Tor variant strains of *V. cholerae* (*ctx*B1 and *ctx*B7)
2013	1. Kashipur, Kalyansingpur, Gunupur, Muniguda, Chandili, Kotapeta, Ramnaguda, Seskhal 2. Dasamantapur, Laxmipur	1. Rayagada 2. Koraput	86	El Tor variant strains of *V. cholerae* (*ctx*B1 and *ctx*B7)
2014	1. Narla	1. Kalahandi	11	El Tor variant strains of *V. cholerae* (*ctx*B7)
2015	1. Majhisahi	1. Nuapada	1	El Tor variant strains of *V. cholerae* (*ctx*B7)
2016	1. Kalyansingpur 2. Komna, Kharsel	1. Rayagada 2. Nuapada	4	El Tor variant strains of *V. cholerae* (*ctx*B7)
2018	1. Ekalabya School 2. Thuamul Rampur	1. Rayagada 2. Kalahandi	5	El Tor variant strains of *V. cholerae* (*ctx*B7)
2019	1. Kalyansingpur	1. Rayagada	31	El Tor variant strains of *V. cholerae* (*ctx*B7)
		Total	520	

### Pulse Field Gel Electrophoresis Analysis

**FIGURE 6:**
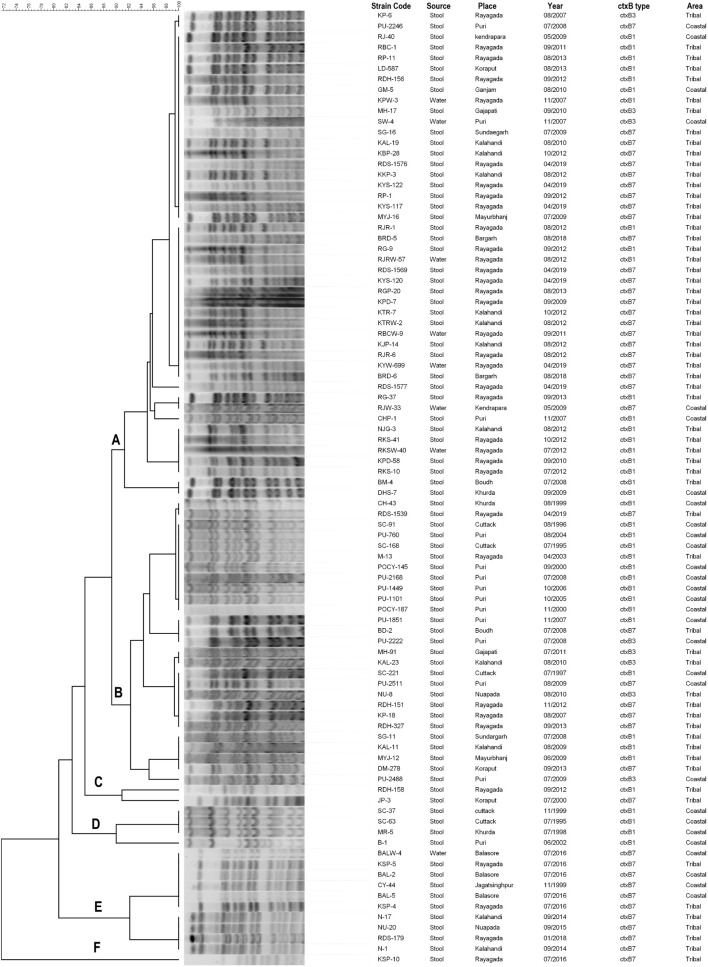
PFGE patterns of the *Not*I-digested *Vibrio cholerae* O1 strains isolated from different districts of Odisha: 1995–2019. Capital letters means different clusters.

A PFGE analysis of *Not*I digested genomic DNA of 90 representatives of *V. cholerae* O1 strains isolated from stool and environmental water sources from different regions of Odisha over the past two and half decades were performed. The majority of strains (*n* = 73) belonged to two major clusters A and B, while the rest of the strains (*n* = 17) showed discrete patterns and thus belonged to four different banding patterns C, D, E, and F (Figure 6). Therefore, it is evident that the spread of El Tor variant strains of *V. cholerae* O1 throughout Odisha might have originated from a single clone or developed from multi-clonal emergence of the El Tor variants of *V. cholerae* O1 in each region of Odisha.

#### Cluster A

Cluster A includes 46 strains of *V. cholerae* O1 that showed close relatedness among the clinical and environmental samples, as the PFGE banding patterns were highly homogenous (100% similarity). For example, the environmental water isolates of *V. cholerae* O1 from Rayagada (KPW-3/2007) possessing the El Tor variant *ctx*B1 genotype exhibited 100% similarity with Rayagada and Kalahandi outbreak strains of 2012 carrying both *ctx*B1 and *ctx*B7 genotypes and also showed similar banding patterns with Bargarh and Rayagada outbreak strains of 2018 and 2019, respectively, where both *ctx*B7 genotypes were reported (Figure 6). It was also interesting to note that typical El Tor strains carrying the *ctx*B3 genotype had a close relationship with El Tor variant strains of *ctx*B1 and *ctx*B7 genotypes. The genetic relatedness linking the coastal and tribal strains of *V. cholerae* O1 was also proved by a 95% similarity coefficient obtained between isolates from these districts.

#### Cluster B

Cluster B includes 27 strains of *V. cholerae* O1 that showed a 90% similarity coefficient with cluster A. This cluster showed an interrelationship between *ctx*B3 with *ctx*B1 and *ctx*B7 genotypes. The *V. cholerae* O1 strains from Puri, Kalahandi, Nuapada, and Gajapati possessed the *ctx*B3 genotype, i.e., prototype El Tor strain of *V. cholerae* O1 characteristic, which exhibited 92% similar banding patterns with *V. cholerae* O1 strains of altered El Tor genotypes isolated from the coastal as well as tribal districts of Odisha from 1995 to 2019. This might indicate that mutation of prototype El Tor strains occurred in due course of time that produced varied genotypes but showed nearly similar banding patterns.

#### Clusters C and D

The clusters C and D showed discrete banding patterns composed of six different strains of *V. cholerae* O1.

#### Cluster E

Cluster E showed an interesting result that comprised 10 *V. cholerae* O1 strains of *ctx*B7 genotype isolated from both the coastal and tribal areas of Odisha that showed the first reported Haitian variant strain (CY-44/1999) having a close link with outbreak strains of Balasore and Rayagada (2016) strains and 94% similarity with Narla outbreak strain of Kalahandi district (2014) and Nuapada (2015) strain. The PFGE analysis of El Tor variant *V. cholerae* O1 strains from Odisha proved that each strain has clonality with the other as more than 80% similarity index was obtained between them.

## Discussion

Cholera has been a major public health concern in the coastal areas in both Bangladesh and India for centuries (Ali et al., 2015). Odisha being a coastal state of India has faced recurrent cholera outbreaks/epidemics followed by cyclone/flood almost every year causing significant morbidity and mortality since 1995–2019 (Nayak et al., 2021). Transmission of cholera in the coastal communities is getting higher due to increased global temperature and the saline-rich water that favors *V. cholerae* and other pathogenic bacteria to flourish and spread across boundaries (Colwell, 2004). At present, altered El Tor strains of *V. cholerae* O1 that were identified in the isolates from 1995 onward replaced the normal prototypes of El Tor strains causing almost all cholera outbreaks and epidemics throughout Odisha. Our earlier studies proved that *V. cholerae* O1 strains isolated from 1995 to 2006 were El Tor variant strains with a *ctx*B classical genotype that had been circulated in Odisha (Pal et al., 2010). However, hybrid El Tor variant strains of *V. cholerae* O1 were also reported from Odisha in 2008 and 2009 that possessed *ctx*B genes of both classical and El Tor biotypes (Khuntia et al., 2013). The hybrid strains of *V. cholerae* O1 have also been reported from other parts of India and Thailand (Taneja et al., 2009; Na-Ubol et al., 2011).

The altered biotype of *V. cholerae* O1 El Tor (classical CT) strains belonged to the Gulf Coast of the United States, designated as genotype 1 (Olsvik et al., 1993). Later the altered El Tor strains carrying classical CT have been reported from different regions of the globe particularly Bangladesh, Mozambique, Vietnam, Hong Kong, Japan, Zambia, India, Sri Lanka, Africa, Nigeria, and Haiti (Nair et al., 2002; Raychoudhuri et al., 2009; Safa et al., 2010; Marin et al., 2013). In Odisha, these strains were first reported in 1995 from Cuttack and later found from other coastal districts, namely, Jagatsinghpur, Kendrapara, Jajpur, Puri, Bhadrak, and Balasore, during the 1999 super cyclone. The data also supported the fact that *V. cholerae* O1 El Tor variant strains were circulating in the tribal districts of Odisha subsequent to the 2007 cholera epidemic (Pal et al., 2010). Previously, changes in the amino acid sequences in *ctx*B of El Tor strains at positions 39 and 68 had been reported and these sequences were similar to those of classical strains (Kumar et al., 2009). However, the emergence of new El Tor variant strains with a modified classical CT due to mutation at amino acid position 20 (histidine–asparagine) has been described as Haitian variant reported elsewhere (Naha et al., 2012). This Haitian *ctx*B strain arose in fame after causing a devastating epidemic in Haiti in 2010. The altered El Tor strains carrying the *ctx*B7 genotype (HCT variant) were first reported from Odisha in 1999 much before the outbreak in Haiti in 2010 (Pal et al., 2017); subsequently reported in Kendrapara district in 2009 and spread to tribal areas of Rayagada and Kalahandi districts in 2012–2019 (Pal et al., 2013, 2017, 2021; Nayak et al., 2020). This strain was also reported from sporadic cholera cases in Kolkata and Yavatmal (Kutar et al., 2013; Kumar et al., 2014); in Africa and Yemen during 2015–2017 (Weill et al., 2019). In Odisha, there is a progressive increasing trend of antibiotic resistance toward commonly used antibiotics such as ampicillin, streptomycin, neomycin, nalidixic acid, co-trimoxazole, and furazolidone, which were used as the first line of treatment for cholera (Nayak et al., 2021). Similar findings have been reported in Ghana and the Democratic Republic of Congo (Miwanda et al., 2015; Abana et al., 2019). Tetracycline-resistant strains of *V. cholerae* O1 were reported in 2010 from the tribal areas of Odisha, but its reversal was observed in successive years (Pal et al., 2018). This phenomenon might be due to the extensive use of tetracycline or due to rapid fluctuations in nature.

In this study, we found that 1,380 isolated *V. cholerae* O1 serotype Ogawa strains had dominance (92.4%) over serotype Inaba (7.6%) and were responsible for all the cholera outbreaks reported from 1999 to 2019. The pathogenesis of cholera is conferred due to synergistic actions of core CTX elements and the TCP pathogenicity island (Faruque et al., 1998). All isolates in this study belonged to the El Tor biotype on the basis of the repeat in toxin gene (*rtxC*). The correlation between the virulence and accessory genes showed positive values except for *rfb*O1. The positive correlation indicated a stronger association between the genes that regulate the action of major toxin production in all the strains. It was noted that VAGS were non-uniformly distributed among *V. cholerae* O1 strains by exhibiting discrete clusters indicative of different virulence patterns shown by these strains.

Analysis of the *Not*I-digested PFGE profiles of *V. cholerae* O1 isolates revealed six different clusters with a similarity matrix of 72%. Among the clusters, clusters A and B comprised 73 strains of *V. cholerae* O1 from the clinical and environmental water sources that shared 92% similarity coefficient among them. This suggests that contamination of the water sources by this pathogen thus might have acted as a reservoir in the transmission of disease (Adewale et al., 2016). It was already published by us that the HCT variant of *V. cholerae* O1 was reported in 2009 (pond water, roadside reservoir water, well water), 2011 (open well), 2012 (stream water), 2016 (open well), and 2019 (Chua water) from both coastal and tribal areas of Odisha. These findings strengthened the fact that the HCT variant of *V. cholerae* O1 had adapted to various environmental water sources to enable viability in different parts of Odisha (Pal et al., 2021). Similar findings were reported from Malaysia in 2009 and also from Bangladesh in 2015–2016, where PFGE results showed clonality among *V. cholerae* strains (Ang et al., 2010; Rahman et al., 2018). Clonality among altered El Tor strains carrying classical *ctx*B1 and Haitian *ctx*B7 genotypes isolated from different geographical regions in Odisha was also proved through PFGE. This finding is similar to the previous reports obtained from Nigeria, Africa, in 2016, where a clonal relationship between classical *ctx*B and Haitian *ctx*B was established through PFGE (Adewale et al., 2016). A pulsotype with 100% similarity index was obtained between the first Haitian variant (*ctx*B7) of *V. cholerae* O1 isolated in 1999 and cholera outbreak strains in 2014–2016 and 2018, respectively, indicative of one clone or lineage of single ancestral origin. Similar findings on the spread of Haitian variant *V. cholerae* O1 strains were reported from South India (Bhattacharya et al., 2015).

In another study, we have detected the environmental reservoirs of *V. cholerae* in the flowing freshwater environs in the tribal areas of Odisha (Pal et al., 2021), where partial stagnant conditions of water at the bank of the river, nala, stream, or temporary storage of water partially encircled by the stones served as the reservoir of *V. cholerae* strains in the flowing aquatic environment which subsequently behaved as the source of infection in the tribal areas. So, as the people in the tribal areas depend on the stream, nala, chua, and river water, the stored water in the hilltops supplied to the villages should be chlorinated in different time intervals before and during the monsoon season. People should be aware toward the use of potable water for drinking and cooking. So, the possible outbreak of cholera will be checked in this region. Previous publications by sequencing and comparing hundreds of bacterial genomes of *V. cholerae* have shown that all the explosive epidemics of cholera in Africa and America in the past-half century arose after the arrival of new strains that had evolved in Asia (Domman et al., 2017; Weill et al., 2017; Pal et al., 2019). The present findings also strengthened the above facts.

## Conclusion

The spread of multidrug-resistant, *ctx*B1- and *ctx*B7-possessing *V. cholerae* O1 strains from the coastal to tribal areas in Odisha occurred during the past two and a half decades in a sequential manner which might be due to a single clone or due to lineages of a single ancestral origin. This study also provides evidence for clinical and water isolates that shared genetic linkage with a similarity matrix of 100% proved through PFGE analysis indicative of environmental water sources which might have acted as a reservoir for the transmission of this disease from the coastal to tribal areas of Odisha. So, provision of potable water supply should be in places especially in communities residing in the inaccessible areas which mainly depend on chua, nala, and stream water particularly in the tribal areas. From the present findings, it is also evident that the altered El Tor *V. cholerae* O1 carrying *ctx*B1 and *ctx*B7 genotypes originated in the east coast of the Bay of Bengal and gradually spread to the tribal areas, which strengthened the hypothesis that the hometown of cholera was the Gangetic belts of Bay of Bengal.

## Data Availability Statement

The original contributions presented in the study are included in the article/supplementary material, further inquiries can be directed to the corresponding author/s.

## Author Contributions

BP conceptualized and designed the study, interpreted the data, and finalized the manuscript. DB analyzed the data and edited the draft for the manuscript. SN and AN did the molecular works and contributed to the data analysis. All authors reviewed and approved the manuscript.

## Conflict of Interest

The authors declare that the research was conducted in the absence of any commercial or financial relationships that could be construed as a potential conflict of interest. The reviewer PK declared a shared affiliation with the authors to the handling editor at the time of the review

## Publisher’s Note

All claims expressed in this article are solely those of the authors and do not necessarily represent those of their affiliated organizations, or those of the publisher, the editors and the reviewers. Any product that may be evaluated in this article, or claim that may be made by its manufacturer, is not guaranteed or endorsed by the publisher.
